# A case report of primary pulmonary artery intimal sarcoma

**DOI:** 10.1186/s40001-021-00568-w

**Published:** 2021-08-09

**Authors:** Xiaofang Bai, Litao Ruan

**Affiliations:** The Department of Ultrasound Medicine, The First Affiliated Hospital, Xi’an Jiaotong University, No. 277, Yanta West Road, Xi’an, 710061 Shaanxi China

**Keywords:** Pulmonary artery intimal sarcoma, Echocardiography, Adult

## Abstract

**Background:**

Pulmonary artery intimal sarcoma (PAS) is a very rare disease, its prevalence is about 0.001–0.003%. PAS is often misdiagnosed as acute or chronic pulmonary thromboembolism due to its clinical presentation and radiological findings. Thus, early diagnosis is very crucial and may improve patient outcome.

**Case presentation:**

Here, we report a case in a Chinese male where the symptom presentation was episodes of shortness of breath. Transthoracic echocardiography showed a solid mass in the pulmonary valve orifice, which was demonstrated to be a pulmonary artery intimal sarcoma diagnosed by histopathology. In this case, the initial differential diagnosis included pulmonary embolism. Because the initial symptom of primary pulmonary artery sarcoma is extremely similar to the pulmonary embolism, half of them may be misdiagnosed as pulmonary embolism. Imaging studies are very helpful. Ultrasound and CT are the best due to their resolution and ability to assess the relationship of the mass with the surrounding structures. The final diagnosis is mostly made after surgical excision and this is the most effective treatment. At the same time, radiotherapy and chemotherapy after surgery is also an adjuvant treatment.

**Conclusion:**

We report a very rare case of pulmonary artery intimal sarcoma. Due to late diagnosis and delayed treatment in this case, the patient displayed a poor prognostic. Early diagnosis and right treatment can improve the prognosis of PAS and optimize overall health.

## Background

Angiosarcoma is a malignant tumor derived from vascular endothelial cells. It has low incidences. It can occur in the skin, breast, spine, small intestine, spleen, kidney and so on, accounting for about 2% of all soft tissue sarcoma. Pulmonary artery sarcoma belongs to one of angiosarcoma, which originates from primitive pluripotent interstitial cells with multiple differentiation ability. Pulmonary artery sarcoma can originate from the left and right pulmonary arteries and intimal layer of pulmonary arteries, forming a tumor growing in the nodular cavity, or spreading along the intimal surface, and retrograde can involve the pulmonary valve and the right ventricle. It can be found in pulmonary artery trunk or unilateral or bilateral pulmonary artery [1].

Pulmonary artery intimal sarcomas are rare. Nonetheless, approximately 400 cases of pulmonary artery intimal sarcomas have been reported in the literature up to the year 2021. Most of them were published by case reports. Because the primary pulmonary artery intimal sarcomas were often misdiagnosed, the incidence of it may be underestimated.

## Case presentation

**Fig. 1:**
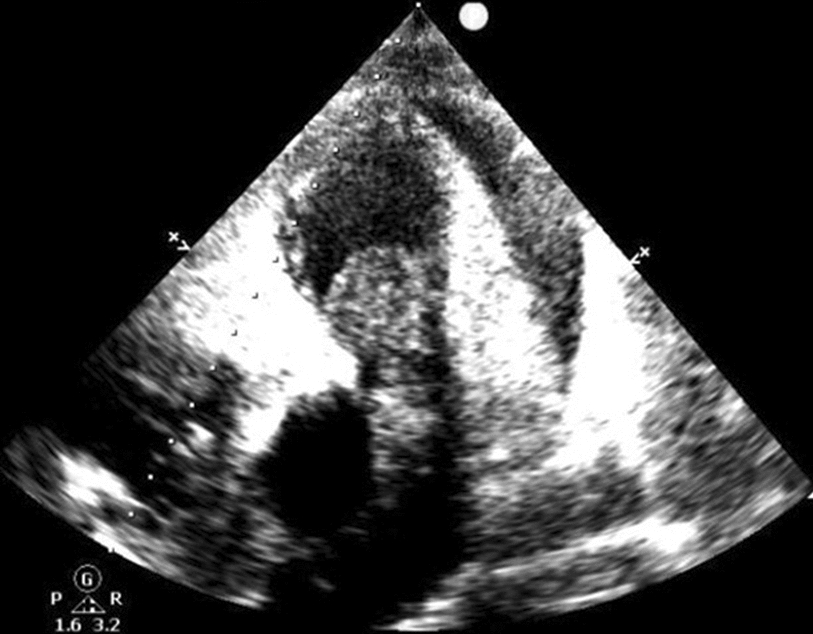
Long axis of pulmonary artery of transthoracic echocardiography. Arrow indicates the location of the lesion

**Fig. 2:**
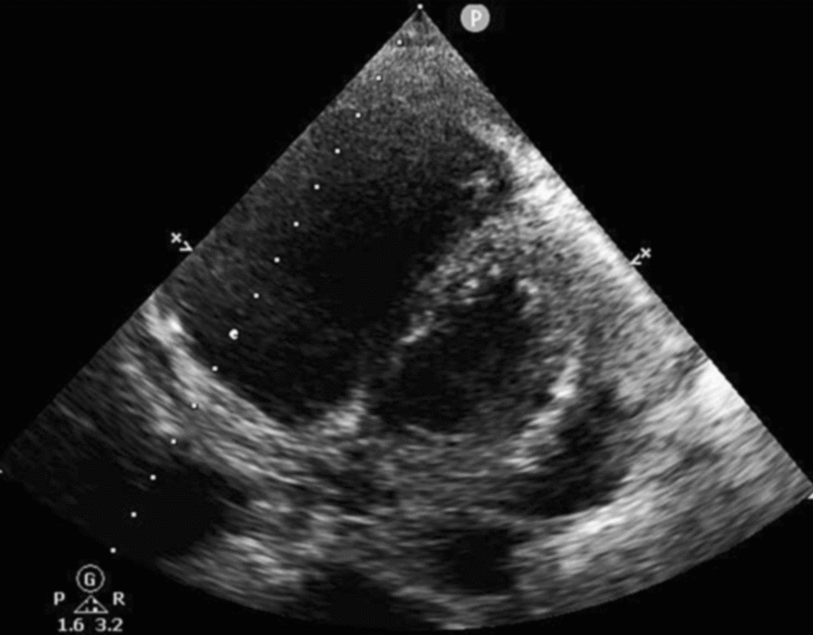
Short axis of left ventricular of transthoracic echocardiography

**Fig. 3:**
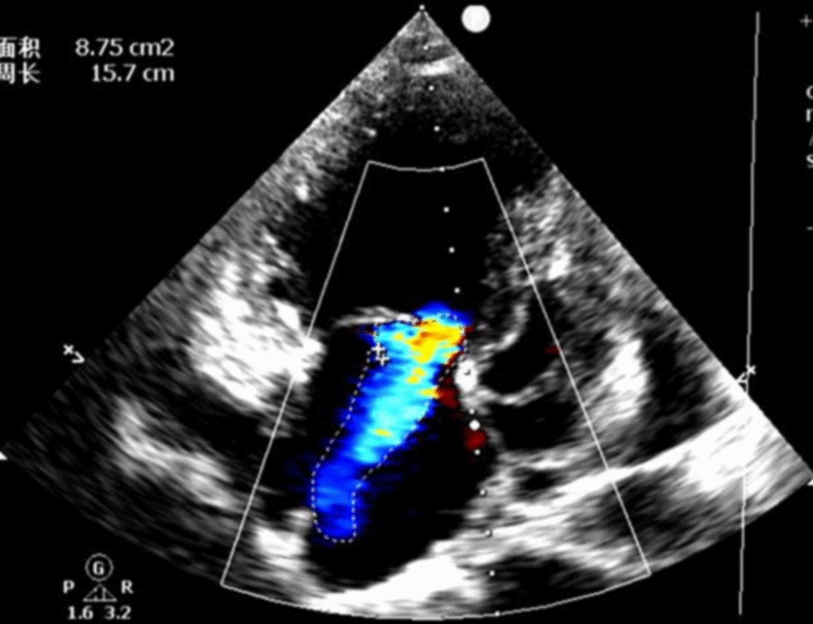
Severe tricuspid regurgitation on color flow Doppler

**Fig. 4:**
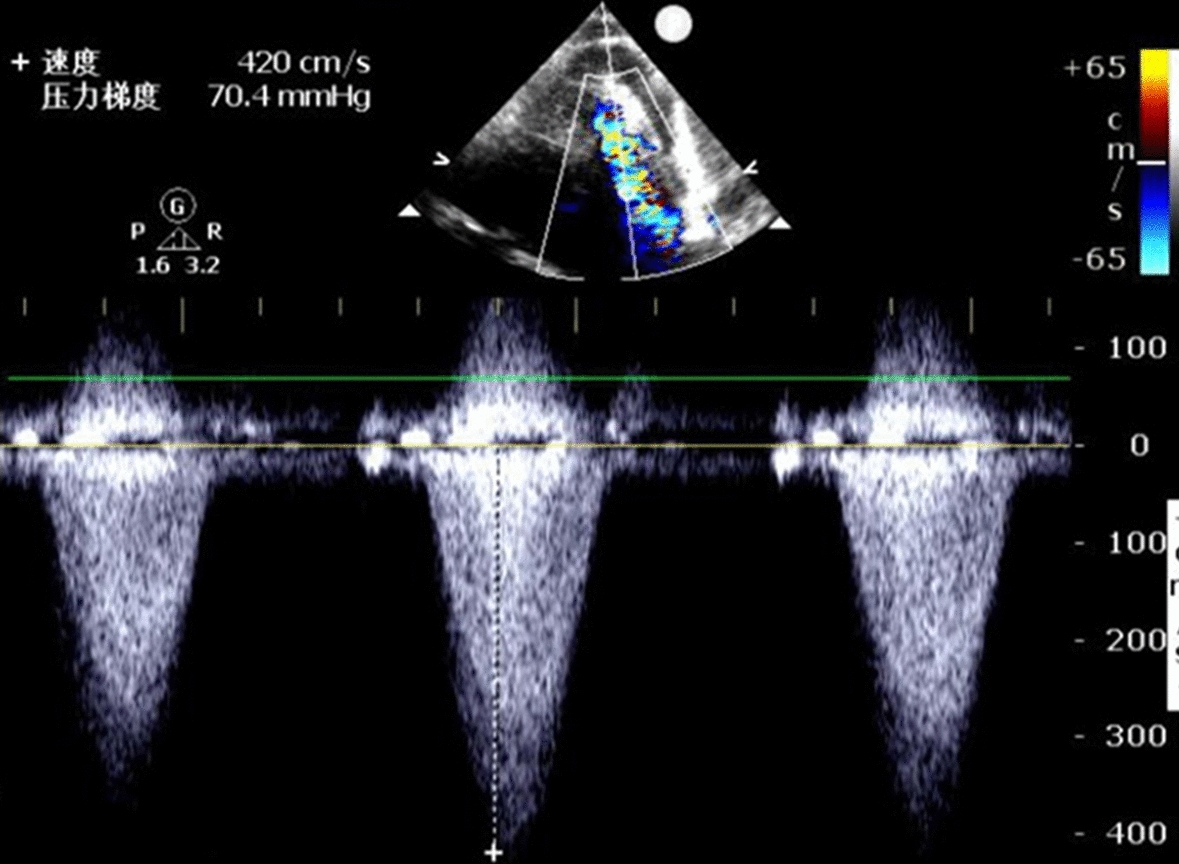
Severe tricuspid regurgitation on spectral Doppler

**Fig. 5:**
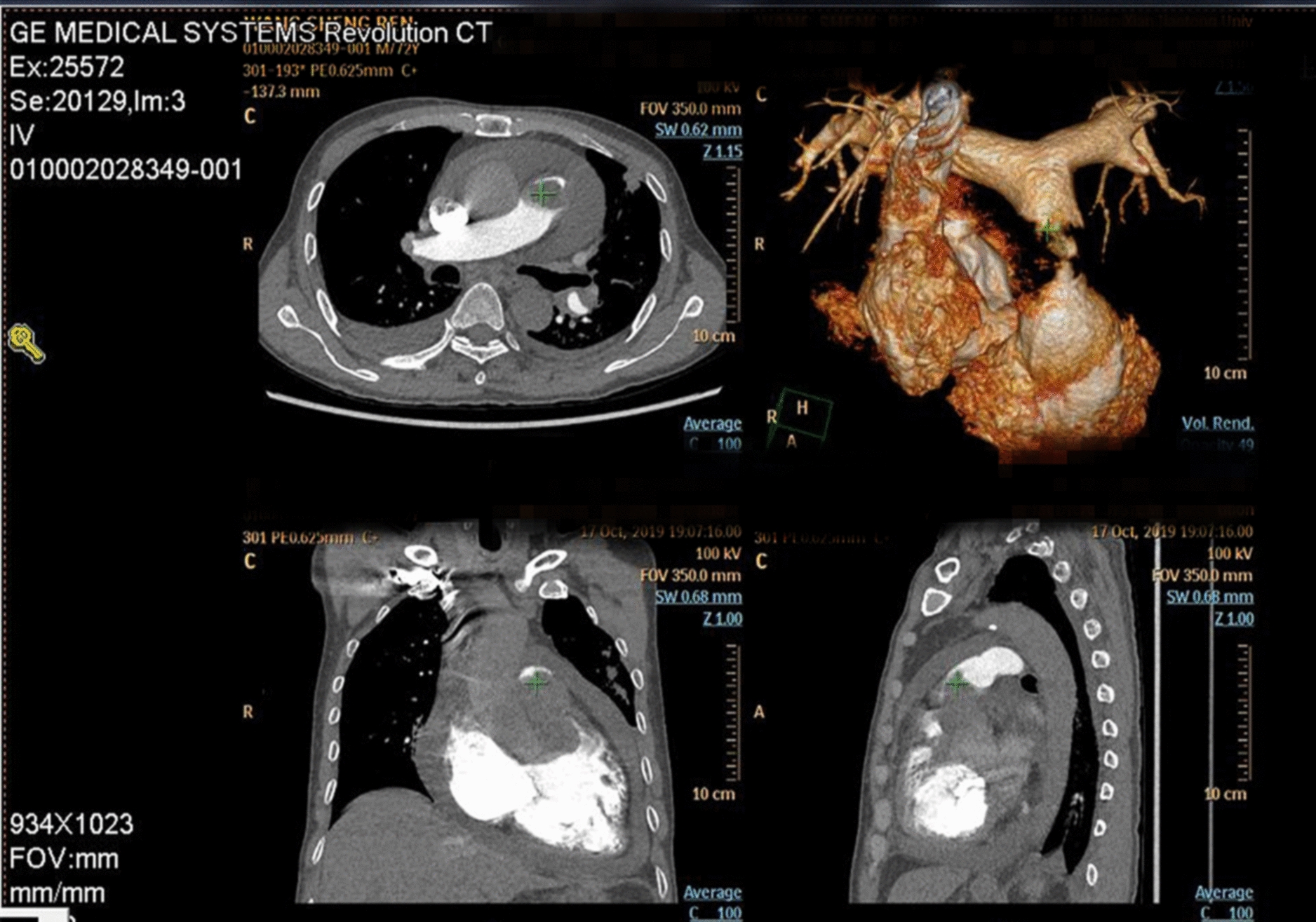
Computed tomography angiography (CTA) findings of superior vena cava images acquired in multiple planes. Arrow indicates the location of the lesion

**Fig. 6:**
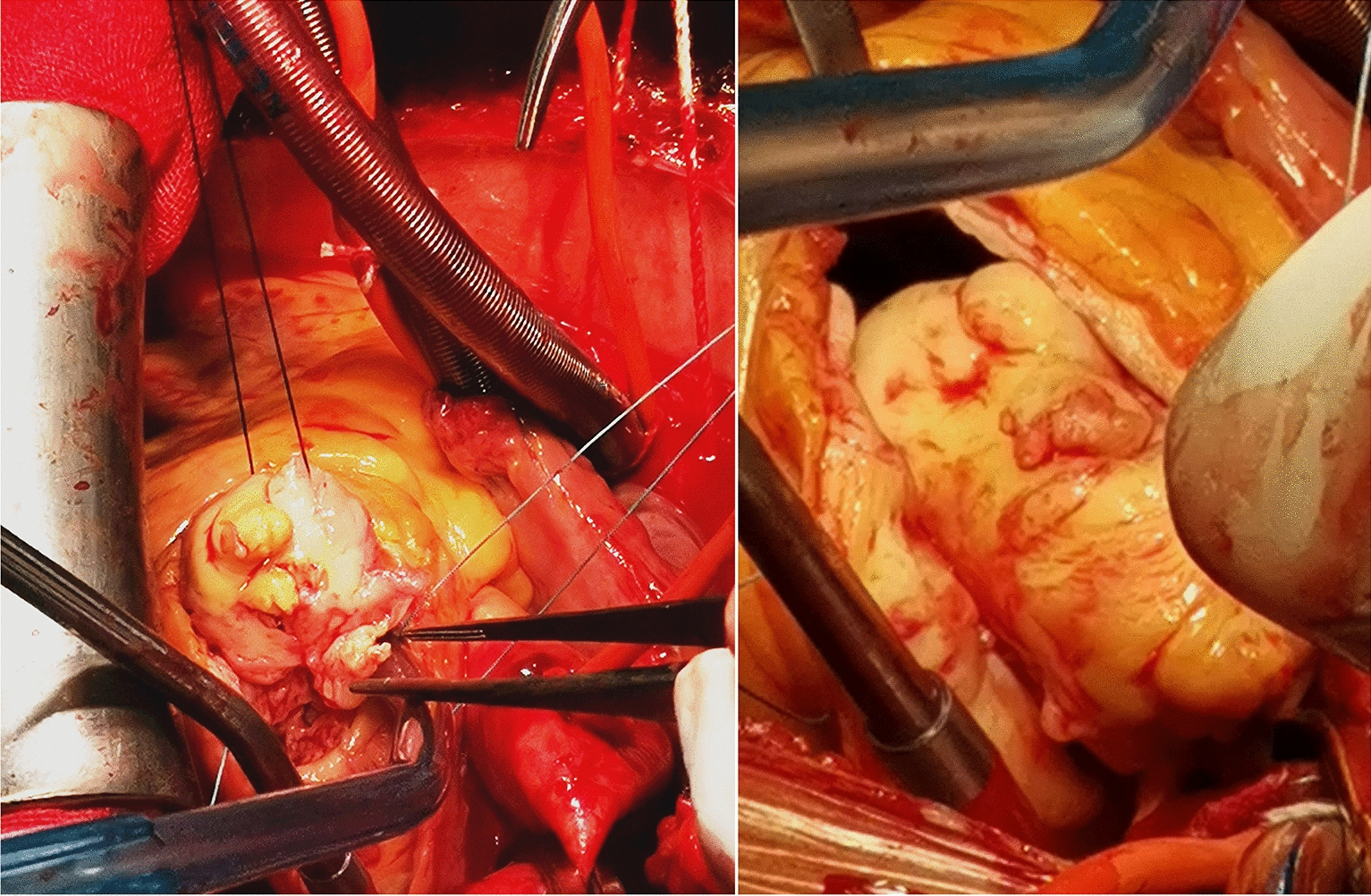
The intraoperative images. Operative view of the primary pulmonary artery intimal sarcoma

**Fig. 7:**
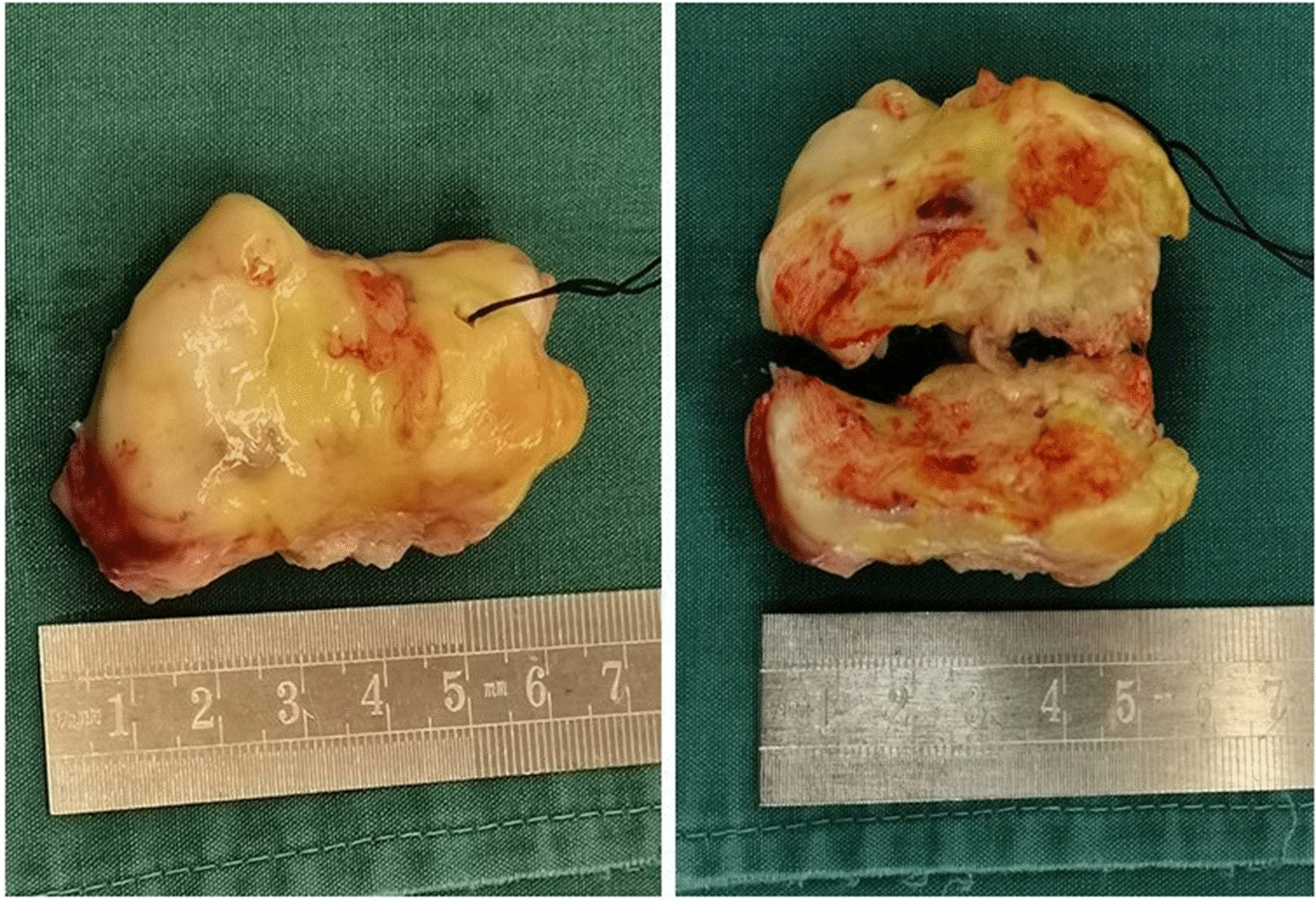
Gross specimen of primary pulmonary artery intimal sarcoma

**Fig. 8:**
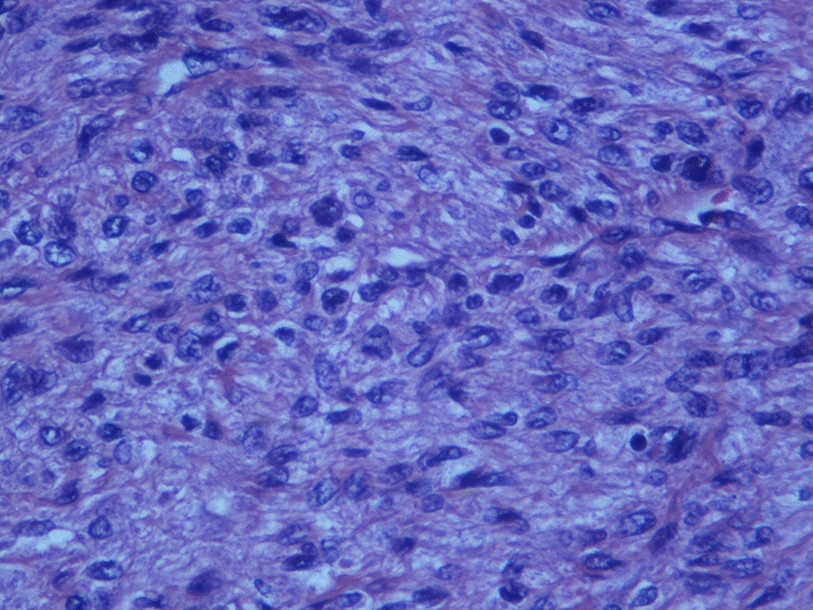
Histopathology of primary pulmonary artery intimal sarcoma

A 72-year-old man was admitted with shortness of breath for more than 1 year after the activity and then had hemoptysis for 1 week. The patient has a history of dermatophytosis for 4 years and never-treated, smoking more than 20 years cigarettes 3 per day and quitting smoking for 6 months, drinking for more than 10 years. On physical examination, her bilateral zygomatic and lips had cyanosis, hepatic jugular venous reflux sign was positive. Mild systolic murmur of grade 2/6 could be heard in the auscultation area of pulmonary valve and peripheral oxygen saturation was 97% while breathing ambient air. Two-dimensional transthoracic echocardiography showed a solid mass was detected at the pulmonary valve orifice, showing moderate-to-strong echo. Its outline was clear, and the internal echo was uniform, with the size of about 57mm × 36mm. It was attached to the pulmonary valve orifice, part of which was located in the right ventricular outflow tract, and part in the main pulmonary artery. There was no obvious motion, causing obvious stenosis of the pulmonary valve orifice (Fig. 1, arrows). The right atrium and right ventricle were enlarged, and the interventricular septum shifted to the left ventricle, showing “D” sign (Fig. 2). Color Doppler flow imaging showed moderate regurgitation in tricuspid valve with regurgitation area of 8.8cm^2^ (Fig. 3), regurgitation velocity of 420 cm/s and PG of 70 mmHg (Fig. 4). Computed tomography angiography (CTA) findings of superior vena cava: right ventricular and the root of pulmonary artery has low-density imaging, considering that it is thrombosis, neoplastic lesions are not excluded (Fig. 5, arrows). The patient underwent surgery, which showed pulmonary valve has solid occupying lesions, pale yellow, soft, nonenveloped, wrapping of the pulmonary valve leaflet, adhesion of the posterior wall of pulmonary valve, clipping the mass along the posterior wall of the pulmonary valve, the size of the mass is about 6.0cm × 4.5 cm (Figs. 6, 7). Cardiac surgeons explored that the posterior wall is very thin, fresh autologous pericardium to reconstruct the pulmonary valve. Continuous observation intraoperative by transoesophageal echocardiography. Postoperative transoesophageal echocardiography demonstrated no obvious abnormalities in the pulmonary arteries. Doppler examination showed a maximum flow velocity of 110 cm/s; color Doppler flow imaging (CDFI) displayed there was no stenosis in the pulmonary arteries. The final pathological diagnosis is Pulmonary artery endometrial sarcoma (Fig. 8). The lesions presented three regions under the microscope: including necrotic regions, sparse region and intensive areas. Tumor cells grow in solid neoplasm, invade from pulmonary artery intima to adventitia, most of the tumor cells are spindle cells, it is very obvious in heteromorphism. Collagen presents predominantly in the interstitial matrix, also bone matrix visible, nuclear compartmentalization and necroptosis is frequent. An immunohistochemical analysis showed that CD vimentin-positive and α-smooth muscle actin-positive, desmin-negative, CD34-negative, CD31-negative, F8-negative, Stat6-negative, Ki67(+ 10%), S100-negative, SOX10-negative, TLE1-negative, CK-negative, EMA-negative. A further treatment for this patient after cardiac surgery was carried on in the cardiac intensive care. Persistent hypoxemia could not correct after giving various intravenous drugs. The patient died in the fifth day after cardiac surgery.

## Discussion and conclusions

### Epidemiology

Large artery endometrial stromal sarcoma is a rare tumor; it is concurrently found in the vein, pulmonary artery, aorta and its branches, pulmonary artery valve, coronary artery, carotid, iliac artery, femoral artery. The incidence of pulmonary artery intimal sarcoma is about 0.001–0.003%. It was first reported by doctor of Mandelstamml in autopsy. The diseases can occur in different age periods, the youngest is 2 months, the oldest is 89 years old, and the average age is about 45–54 years [2].

### Histology and pathology

The correct diagnosis of PAS is based on the pathological examination. According to the origins of the cells, it can be divided into two types, including endometrial type and wall type (originating from intima–media or the outer membrane). Most of the primary sarcoma of aortic and pulmonary artery is endometrial type, arising in pluripotent hematopoietic stem cells of intimal, Conforming to myofibroblastic tumor, with different cell differentiation of different cells. The histological patterns of this tumor ranges from undifferentiated round cell to spindle cell in the light microscope, ten subtypes of this has been reported at least morphologically, including undifferentiated sarcoma, leiomyosarcoma, rhabdomyosarcoma, chondrosarcoma, osteosarcoma, malignant phyllodes tumor, fibrosarcoma, myxosarcoma, angiosarcoma, malignant fibrous histiocytoma. Most of the pulmonary artery intimal sarcoma is not easy to classify, it is briefly defined as intimal sarcoma or undifferentiated sarcoma [3].

### Clinical presentation

PAS may occur in the main pulmonary artery or in the left and right pulmonary arteries, extending to the distal pulmonary artery. The clinical manifestations were similar to those of pulmonary hypertension and right cardiac insufficiency. In medical imaging, it is easy to be confused with pulmonary arteritis with thrombosis, giant pulmonary thromboembolism and other pulmonary vascular diseases. Common symptoms involve dyspnea, chest pain or back pain, cough, fever, weight loss, hemoptysis, syncope and asthenia [4–6].

### Diagnostic approach

In patients suspected of pulmonary thromboembolism, when radiography shows unilateral pulmonary artery dilatation, persistent soft tissue filling pulmonary artery, pulmonary mass after anticoagulant and thrombolytic therapy, ultrasound found that the pulmonary artery dilatation, irregular mass, uneven echo, lobular shape, extraluminal invasion, all highly suggested the disease. Color Doppler flow imaging shows the relationship between pulmonary artery sarcoma and pulmonary artery and left and right pulmonary arteries in real time. Two-dimensional ultrasound imaging can show the heterogeneous parenchymal echo of pulmonary artery sarcoma, which is helpful to distinguish it from the homogeneous and weak echo of fresh thrombus of pulmonary embolism, color and spectrum Doppler can dynamically show the relationship between blood flow and tumor echo in stenosis, and can detect blood velocity. Color echocardiography is of great value in the diagnosis, differential diagnosis and postoperative follow-up of pulmonary artery sarcoma.

### Treatment

At present, it is agreed that surgical resection of tumor is the first choice of treatment for pulmonary intimal sarcoma, To completely clear the lesion, this method can prolong the survival time; The role of radiotherapy and chemotherapy is controversial, but there is a tendency for surgery combined with chemotherapy and/or radiotherapy, especially for those who cannot complete surgical resection or recurrence after surgery, more recommended to try; 20% of patients responded better to chemotherapy or radiotherapy. Prognosis of primary pulmonary intimal sarcoma is poor; tumor can be transferred to brain, pancreas, adrenal gland, lung. A life expectancy of 12–18 months after symptoms, the 1-year and 2-year survival rates were 22 and 7%, respectively. The median survival of patients unable to operate due to disease progression (progressive right heart failure) was only 6 weeks, the survival time of patients with surgical resection can be up to 3 years [7–17].

## Conclusion

Pulmonary artery intimal sarcoma is similar to the diseases of thromboembolism clinically. It is very important to differentially diagnose these two diseases because of the different treatment options and prognosis. Pathological diagnosis is the ultimate diagnosis.

## Data Availability

All data generated or analyzed during this study are included in this published article.

## Abbreviations

PAS: Pulmonary artery intimal sarcoma
CTA: Computed tomography angiography
CDFI: Color Doppler flow imaging

## References

[1] Xu R, Zhao Y, Xu X (2020). Pulmonary intimal sarcoma involving the pulmonary valve and right ventricular outflow tract: a case report and literature review. Medicine.

[2] Ding-Yu C (2020). Pulmonary artery intimal sarcoma: a case report and literature review. Respirol Case Rep.

[3] Ushioda R, Kitahara H, Ise H (2019). A case of pulmonary artery sarcoma that was initially mis-diagnosed as pulmonary embolism. J Surg Case Rep.

[4] Yeungd F, Johnston A, Simmons C (2018). Multimodality imaging of a pulmonary artery sarcoma. Echocardiography.

[5] Lee Y, Kimh J, Yoon H (2016). Clinical characteristics and treatment outcomes of primary pulmonary artery sarcoma in Korea. J Korean Med Sci.

[6] Drozdz J, Warcho E, Fijuth J (2013). Primary pulmonary artery sarcoma in 36-year-old women: 3-years follow-up after partial resection and radiotherapy. Kardiol Pol.

[7] Long HQ, Qin Q, Xie CH (2008). Response of pulmonary artery intimal sarcoma to surgery, radiotherapy and chemotherapy: a case report. J Med Case Rep.

[8] Wilkens H, Konstantinides S, Lang I (2016). Chronic thromboembolic pulmonary hypertension: recommendations of the cologne consensus conference 2016. Dtsch Med Wochenschr.

[9] Srivali N, Yie S, Ryu JH (2017). Pulmonary artery sarcoma mimic king pulmonary embolism: a case series. QJM.

[10] Yin K, Zhang Z, Luo R (2018). Clinical features and surgical outcomes of pulmonary artery sarcoma. J Thorac Cardiovasc Surg.

[11] Deng L, Zhu J, Xu J (2018). Clinical presentation and surgical treatment of primary pulmonary artery sarcoma. Interact Cardiovasc Thorac Surg.

[12] Yamamoto Y, Shintani Y, Funaki S (2018). Aggressive surgical resection of pulmonary artery intimal sarcoma. Ann Thorac Surg.

[13] Wyler Vonballmoos MC, Chan EY, Reardon MJ (2019). Imaging and surgical treatment of primary pulmonary artery sarcoma. Int J Cardiovasc Imaging.

[14] Morreau SP, Haydock DA (2017). Prolonged survival of pulmonary artery sarcoma after aggressive surgical resection. Ann Thorac Surg.

[15] Jenkins DP, Madani M, Mayer E (2013). Surgical treatment of chronic thromboembolic pulmonary hypertension. Eur Respir J.

[16] Gan HL, Zhang JQ, Zhou QW (2011). Surgical treatment of pulmonary artery sarcoma. J Thorac Cardiovasc Surg.

[17] Yamasaki M, Sumi Y, Sakakibara Y (2011). Pulmonary artery leiomyosarcoma diagnosed without delay. Case Rep Oncol.

